# Prevalence of antenatal depression and its association with adverse childhood experiences: a cross- sectional study at Hospital Canselor Tuanku Muhriz, Kuala Lumpur

**DOI:** 10.3389/fpsyt.2025.1574094

**Published:** 2025-09-03

**Authors:** Lalijah Nagenthiran, Fairuz Nazri Abd Rahman, Raynuha Mahadevan, Ani Amelia Zainuddin, Rosnah Sutan

**Affiliations:** ^1^ Department of Psychiatry, Hospital Kajang, Selangor, Malaysia; ^2^ Department of Psychiatry, Faculty of Medicine, Hospital Canselor Tuanku Muhriz, Kuala Lumpur, Malaysia; ^3^ Child and Adolescent Psychiatric Unit, Faculty of Medicine, UKM Specialist Children’s Hospital, Kuala Lumpur, Malaysia; ^4^ Department of Obstetrics and Gynaecology, Faculty of Medicine, Hospital Canselor Tuanku Muhriz, Kuala Lumpur, Malaysia; ^5^ Department of Community Health, Faculty of Medicine, Hospital Canselor Tuanku Muhriz, Kuala Lumpur, Malaysia

**Keywords:** antenatal, depression, adverse childhood experiences, maternal mental health, childhood trauma

## Abstract

**Introduction:**

Emerging evidence suggests a link between adverse childhood experiences (ACEs) and antenatal depression, with a higher number of maternal ACEs associated with increased depressive symptoms during pregnancy.

**Objective:**

This study aimed to assess the prevalence of antenatal depression and its association with ACEs among pregnant women in a hospital setting. Methods: A cross-sectional study was conducted among 314 pregnant women recruited through convenience sampling between 18th November 2024 to 31st December 2024. Inclusion criteria were pregnant women aged 18 years and older, able to communicate in English or Malay, able to consent, and Malaysian citizens. Data collection involved self- reported sociodemographic questionnaire, the Edinburgh Postnatal Depression Scale (EPDS) and Adverse Childhood Experiences– International Questionnaire (ACE -IQ). Participants were stratified by ACE score groupings (0, 1, 2, 3, ≥4), and associations with depression risk were analyzed using logistic regression models, adjusting for confounders such as race, occupation, planned pregnancy, and trimester. The association between specific ACE domains and depression risk was also explored.

**Results:**

The prevalence for antenatal depression was 13.7%. with 70.4% reporting at least one ACE. Higher ACE scores were significantly associated with increased depression risk. Women with four or more ACEs had more than a sevenfold increased odds of depression (OR = 7.3, 95% CI = 2.79, 18.71, p < 0.001). In adjusted models, the association persisted (aOR = 4.60, 95% CI = 1.32, 16.02, p = 0.017). Specifically, physical abuse (aOR = 5.08, 95% CI = 2.20, 11.73, p < 0.001), emotional abuse (aOR = 3.54, 95% CI = 1.66, 7.58, p = 0.001), household member treated violently (aOR = 4.400, 95% CI = 1.94, 9.96, p < 0.001), physical neglect (aOR = 2.65, 95% CI = 1.26, 5.59, p = 0.010), and community violence (aOR = 2.16, 95% CI = 1.08, 4.33, p = 0.030) were all significantly related to depression risk.

**Conclusion:**

Our findings suggest that ACEs are strongly associated with an increased risk of depression among pregnant women and highlights the importance of integrating depression and ACE screening into antenatal services.

## 1 Introduction

Pregnancy is a unique maternal experience with profound biological, psychological, and social impacts on women. Nearly one-fifth of pregnant women experience mental health issues during the perinatal periods (1). Depression is particularly prevalent, affecting about 13% of pregnant women, making it the second leading cause of disability among women worldwide (2). Among these conditions, antenatal depression stands out as one of the most common psychiatric disorders, with serious consequences for both maternal and fetal well- being. Studies found a wide range in the prevalence of antenatal depression globally, including 31.1% in Ethiopia, 20.7% in Turkey, and 8.5% in the United States. Systematic reviews on antenatal depression have provided useful insights, yet there remains a lack of internationally representative prevalence estimates. In Malaysia, a cohort study spanning the east and west coasts found that 12.2% of pregnant women experienced antenatal depression (3). In another study conducted in Kuantan, Pahang, the prevalence rate of antenatal depression was found to be 20.2% (4). The prevalence of antenatal depression was found to be 8.4% in another cross- sectional study which was conducted in Melaka (5). A systematic review showed that any antenatal depression was reported to be 20.7 percent and out of that number, 15.0 percent of pregnant women experienced severe antenatal depression (6). Although the underlying pathophysiology is not fully understood, increase in maternal levels of cortisol have been associated with depression, anxiety, and stress. This excess cortisol can cross the placenta, potentially affecting fetal development. For mothers, antenatal depression is associated with poor nutrition, inadequate weight gain, and increased use of alcohol, tobacco, and other substances (7). For the fetus, the effects of antenatal depression can include stillbirth, preterm birth, low birth weight, low Apgar scores, smaller head circumference, and major congenital anomalies (7). Children of mothers with depression may undergo epigenetic changes linked to future health risks, while altered placental function in these mothers can negatively impact fetal brain development (8). They are at higher risk for neurodevelopmental delays, emotional and behavioral problems, including ADHD and conduct disorders (7). Additionally, these children tend to experience higher rates of anxiety, depression, and substance abuse (9). Antenatal depression is one of the risk factors for the development of postnatal depression, which, in turn, is closely linked to parenting stress and challenges in the mother-infant relationship (10).

One emerging area of research is the role of adverse childhood experiences (ACEs) in the development of antenatal depression. ACEs are defined as stressful or traumatic events that occur during childhood (typically from birth until 18 years) and result in harm or potential for harm to a child’s health, survival, development, or dignity (11). ACEs have lasting impacts on health and well-being, increasing the risk of physical harm, infections, and exploitation. They are also linked to maternal and child health issues, including teen pregnancy, complications, and fetal death (12). The long-term effects of ACEs also contribute to the development of chronic diseases and are linked to leading causes of death, such as cancer, diabetes, heart disease, and suicide (13). From a neurobiological and epidemiological perspective, early exposure to toxic stress, including abuse, can lead to epigenetic changes that alter gene expression (14). These disruptions can result in difficulties with attention, decision-making, and learning, and may lead to structural changes in the brain (14). Such alterations can affect an individual’s brain function, quality of life, and overall health, potentially lasting throughout their lifetime (14). The effects of ACEs can be intergenerational, especially when combined with ongoing trauma like racial discrimination and socioeconomic disadvantage, potentially passing challenges from one generation to the next (15).

One recent national survey in the USA found that between 15.2 and 34.1 percent of women had a history of childhood adversity, demonstrating the high prevalence of ACEs among women (16). In a recent meta-analysis conducted involving 7788 participants with publications from 1998 to 2020, it was found that maternal ACEs are positively associated with antenatal depression and higher number of maternal ACEs was associated with higher reports of depressive symptoms in pregnancy (17). Despite these findings, the literature review reveals a scarcity of studies exploring the relationship between ACEs and antenatal depression in Malaysia. This study aims to examine the prevalence, frequency of various types of ACEs among these patients. Furthermore, the study will explore the association between the different ACEs and antenatal depression, exploring how early-life trauma may contribute to the development of depression during pregnancy. Given the potential impact of these factors, this study hypothesizes that there is an association between the presence of ACEs and antenatal depression. While total ACE scores reflect the number of adversities, they may overlook the frequency or severity of specific experiences. Some categories, such as emotional or physical abuse, may be underrepresented if reported as infrequent. To better capture the full impact of childhood adversity, this study assessed both the number and frequency of ACEs in relation to antenatal depressive symptoms. Through these objectives, this research aims to underscore the importance of routine screening for both antenatal depression and ACEs in pregnant women to better identify and support those at risk.

## 2 Methodology

### 2.1 Study design, setting, and sample

This cross-sectional study was conducted in the Obstetrics and Gynaecology (O&G) Department of Hospital Canselor Tuanku Muhriz HCTM), Cheras, Federal Territory Kuala Lumpur. It is a UKM university hospital under the Ministry of Higher Education, unlike most other hospitals in Malaysia which are under the Ministry of Health. The location of this hospital in Kuala Lumpur, the capital city of Malaysia, is in the central region of the west coast of Peninsular Malaysia. The population of Kuala Lumpur is estimated to be 8,815,630 in 2024. HCTM is situated within the Cheras area of the city. As such, the sample in this study is drawn from a diverse population within Kuala Lumpur, benefiting from the healthcare services provided by HCTM (18). In 2023 alone, more than 10,592 patients attended the antenatal clinic at HCTM. The participants were recruited from both the outpatient clinic and inpatient wards, specifically focusing on pregnant women seeking antenatal care.

The sample size for this study was calculated based on the prevalence of antenatal depression in pregnant women with and without adverse childhood experiences (ACEs) in the USA. Specifically, 14.6% of women with ACEs were reported to experience antenatal depression, compared to 7.29% of those without ACEs (19). Assuming a 95% confidence interval (CI) and aiming for sufficient statistical power, a Type I error probability of 0.05 and a power of 80% were applied, yielding a required sample size of 285 participants. To account for a potential 10% non-response rate, the final sample size was adjusted to 314 participants, ensuring the robustness and reliability of the findings. This sample size calculation was designed to provide meaningful insights into the relationship between antenatal depression and ACEs while maintaining the power to detect significant associations. The inclusion criteria for this study stipulated that participants must be Malaysian pregnant women aged 18 years or older, able to provide informed consent independently, and proficient in reading and understanding either English or Malay. Exclusion criteria were applied to women who were unable to participate in the study due to severe physical or cognitive impairments, active labor, reduced consciousness, or if they were in the patient admission center, labor room or operating theatres during the study period.

### 2.2 Data collection

The sample recruitment for this study was conducted using a convenience sampling method, with data collection taking place between November 18, 2024, and December 31, 2024. Participants were selected from both the Obstetrics and Gynaecology (O&G) Outpatient Clinic and the inpatient O&G ward at HCTM. Outpatient participants were approached during their routine antenatal check-ups, while inpatient participants were approached during their stay in the antenatal ward, either for monitoring of pregnancy-related complications or preparations for delivery.

Each participant was approached individually in a manner that ensured privacy and comfort. Participants were informed about the study’s purpose, procedures, and confidentiality protocols. A Participant Information Sheet (PIS) was provided, and written informed consent was obtained before participation. Upon obtaining consent, participants were given three questionnaires to complete in a private environment. These questionnaires gathered information on sociodemographic details (sociodemographic questionnaire), adverse childhood experiences (ACE-IQ), and depressive symptoms (EPDS), taking approximately 20 minutes to complete. Participants were reminded that they could withdraw from the study at any point without providing a reason and their treatment would not be affected. The data collection process proceeded without any incidents, and participants did not report distress when recalling past experiences while completing the questionnaires.

### 2.3 Ethical approval

The study was conducted in accordance with the ethical guidelines set forth in the Declaration of Helsinki and the Malaysian Good Clinical Practice Guidelines. Ethical approval was granted by the UKM Research and Ethics Committee (UKM PPI/111/8/JEP-2024-214). Additionally, authorization was obtained from the hospital director of HCTM to conduct the study, along with all other necessary approvals before the commencement of the research.

To maintain confidentiality, all data collection sheets were coded and securely stored in a locked cabinet. Hard copies of the study data will be retained for a period of 10 years, after which they will be securely destroyed. Participants identified as being at risk for antenatal depression based on their EPDS score were provided with contact information for the HCTM Psychiatry Clinic for further support. Additionally, information about available mental health services and support were given to participants as part of the ethical considerations of the study.

### 2.4 Study instruments

#### 2.4.1 Malay and English version of the adverse childhood experiences– international questionnaire

The Adverse Childhood Experiences International Questionnaire (ACE-IQ) is a self-report instrument developed by a team led by the World Health Organization (WHO) to explore the connections between adverse childhood experiences and subsequent health outcomes and risk behaviors in individuals aged 18 years and older, worldwide (20). The ACE-IQ consists of 29 items organized into 13 categories: emotional abuse, physical abuse, sexual abuse, violence against household members, living with individuals in the household who are substance abusers, living with mentally ill or suicidal household members, having household members who have been imprisoned, growing up with one or no parents, experiencing parental separation or divorce during childhood, emotional neglect, physical neglect, bullying, exposure to community violence, and exposure to collective violence. The ACE-IQ is recognized for its reliability and validity, with its subscales demonstrating good test-retest reliability with a Cronbach’s alpha of 0.85 (21). Written consent to use this questionnaire was obtained from the WHO.

In Malaysia, the ACE-IQ has been translated into Malay and validated for use among older adults. The translated version showed good internal consistency (Cronbach’s alpha = 0.701), strong content validity confirmed by local experts (Item Content Validity Index I-CVI > 0.8), and acceptable convergent and discriminant validity. Permission to use the translated and validated version of the questionnaire was also obtained from the translator (22).

#### 2.4.2 Malay and English version of Edinburgh postnatal depression scale

The Edinburgh Postnatal Depression Scale (EPDS), originally developed in the United Kingdom, is one of the most widely used screening tools in perinatal care. It is a validated, 10- item, self-reported scale designed to assess both antenatal and postpartum depression on a Likert scale ranging from 0 to 3. Participants are asked to select the response that best reflects their feelings over the past week. The individual item scores are then summed to produce a total score ranging from 0 to 30, with higher scores indicating more severe symptoms (23). Results of a study done by Bergink et al. (2011) showed that the EPDS is a reliable tool for identifying depression in pregnant women. The EPDS’s high concurrent validity and test-retest reliability further supported its generally strong psychometric qualities with Cronbach’s alpha of 0.77 (24). In another systematic review which was done to compare the predictive validity of the Edinburgh Postnatal Depression Scale (EPDS) and other tools for screening depression in pregnant and postpartum women, it was concluded that EPDS can be used in preference to other tools to screen for depression in perinatal women which includes both the antennal and postnatal period (25). The EPDS has also been validated in Malay for use in Malaysia, specifically for screening postpartum depression, with a sensitivity of 72.7% and a specificity of 95% at a cutoff score of 11.5 (26). According to the Malaysian Clinical Practice Guidelines on the Management of Major Depressive Disorder, the cutoff for the Malay version of the EPDS is set at ≥12, which was used as the threshold in this study (27).

#### 2.4.3 Socio-demographic questionnaire

Data collected for the study included sociodemographic information such as age, ethnicity, marital status, highest level of education, occupation, and total monthly household income. Additionally, clinical characteristics were recorded, including whether the participant was an inpatient or outpatient, the number of previous pregnancies, pregnancy planning, trimester, reasons for follow up or hospitalization and the presence of any underlying psychiatric or medical illness as well as any diagnoses related to complications in the current pregnancy.

### 2.5 Statistical analysis

The data were analyzed using the Statistical Package for Social Sciences (SPSS) version 23. Normality was assessed using the Kolmogorov-Smirnov test, which indicated a significant deviation from normal distribution (p < 0.05), prompting the use of non-parametric statistical methods.

All questionnaires were reviewed for completeness, and no missing or out-of-range data were identified. Descriptive statistics were used to examine the distribution of the data. Bivariate analyses, including Chi-square test and independent t-test were conducted to explore associations between independent variables and the dependent variable.

A multivariable logistic regression model was employed to identify factors associated with antenatal depression, with ACEs as the primary predictor. Potential confounders, such as age, race, marital status, education level, occupation, pregnancy planning, trimester, and household income, were included in the model.

To further analyze ACEs, participants were categorized based on the number of ACEs they experienced (none, 1, 2, 3, or ≥4), and these groups were compared with participants who had no ACEs (reference group). Three distinct models were constructed for adjustment: the first model examined the unadjusted association between ACE score groupings and depression risk; the second model adjusted for the frequency of ACE score groupings; and the third model further adjusted for potential confounders, including age, occupation, pregnancy planning, and trimester. Nagelkerke’s R² was calculated for each regression model. Adjusted odds ratios (aORs) and 95% confidence intervals (CIs) were computed, with statistical significance set at p < 0.05 using two-sided tests.

### 2.6 Data transformation

Several variables were recategorized before conducting inferential analyses to address potential imbalances within the groups and to ensure adequate representation in each category, which is crucial for accurate statistical modelling. The “race” variable was simplified into two categories: Malay and Other Races, due to the predominance of Malay participants, which resulted in an imbalance when considering each racial group separately. Occupation was dichotomized into Employed and Unemployed, with the latter group including both unemployed participants and housewives, as housewives were considered part of the unemployed category due to their similar socioeconomic characteristics. Household income was recategorized into five income brackets: < RM2500, RM2501–RM4500, RM4501–RM6500, RM6501–RM8500, and > RM8500. Education level was grouped into two categories: secondary education and below, and higher than secondary education, to contrast individuals with higher education against those with lower educational attainment, as education may influence mental health risks. The number of previous pregnancies was recategorized into five groups: 0, 1, 2, 3, and ≥4 pregnancies, allowing for clearer distinctions between women with fewer and more pregnancies. Finally, the number of Adverse Childhood Experiences (ACEs) was categorized into five groups: 0, 1, 2, 3, and ≥4 ACEs, with participants reporting four or more ACEs grouped together due to the relatively small number in higher ACE categories, ensuring adequate sample sizes for analysis.

## 3 Results

A total of 324 pregnant women were approached for the study. However, 314 were successfully interviewed, resulting in a response rate of 96.9%. Four participants refused to participate, two were excluded as they were non-Malaysian citizens, one was underaged, and the remaining participants were unable to complete the questionnaires due to being called in for doctor’s review or scans.

**Table 1** shows the sociodemographic characteristics of participants at HCTM. A total of 314 pregnant patients participated in this study, with a mean age of 31.6 years (SD = 4.63). The majority of participants were Malay (82.5%), followed by Chinese (9.2%), Indian (3.2%), and others (5.1%), out of which 99.4% were married. In terms of occupation, almost 3 quarter of participants were employed (72.0%) of the participants were employed and 28.0% were unemployed. The distribution of monthly household income showed that the largest group (34.7%) had an income between RM2501 and RM4500 and a smaller proportion of participants had incomes below RM2500 (18.2%), or above RM8501 (13.0%). More than 3/4th had attained at least a college or university level education (76.4%), with 21.7% having completed secondary education, and a small proportion (1.6%) reporting lower education levels. Concerning pregnancy-related characteristics, 23.6% of patients were nulliparous. Fewer patients reported higher numbers of previous pregnancies, with 17.2% having three, 8.3% having four, and smaller proportions reporting five or more. Of the participants, more than half (55.1%) reported that their pregnancies were planned with 79.0% of them being in the third trimester. Almost one third of the participants were being admitted as inpatients and since they were selected using a convenience sampling method which may not fully represent the broader population, statistical analyses will not be conducted comparing inpatient and outpatient to avoid drawing unsupported conclusions. Most hospitalizations (86.0%) were for delivery, with the remaining 14.0% attributed to other illnesses. Regarding medical and psychiatric conditions, 95.0% of patients did not report any major psychiatric illnesses. A small number had a history of major depressive disorder (0.6%), anxiety disorder (2.5%), or multiple psychiatric conditions (1.9%). As for medical comorbidities, 79.6% of the participants were well, while 8.6% had metabolic-related conditions, and another 11.8% reported other medical issues.

**Table 1:** Sociodemographic characteristics of participants at HCTM (n = 314).

Variables	Mean (SD)	n (%)
Sociodemographic Characteristics		
Age (years)	31.6 (4.63)	
Race		
Malay		259 (82.5)
Chinese		29 (9.2)
Indian		10 (3.2)
Others		16 (5.1)
Marital Status		
Single		2 (0.6)
Married		312 (99.4)
Occupation		
Employed		226 (72.0)
Unemployed		88 (28.0)
Monthly Household Income		
< RM2500		57 (18.2)
RM2501–RM4500		109 (34.7)
RM4501–RM6500		63 (20.1)
RM6501–RM8500		44 (14.0)
RM8501–RM10500		23 (7.3)
> RM10500		18 (5.7)
Education Level		
No formal education		1 (0.3)
Primary		4 (1.3)
Secondary		68 (21.7)
College or University		240 (76.4)
Others		1 (0.3)
Pregnancy and Clinical Characteristics		
Number of Previous Pregnancies		
0		74 (23.6)
1		72 (22.9)
2		66 (21.0)
3		54 (17.2)
≥4		48 (15.3)
Planned Pregnancy		
No		141 (44.9)
Yes		173 (55.1)
Trimester		
First		13 (4.1)
Second		54 (17.2)
Third		247 (78.7)
Patient Status		
Inpatient		92 (29.3)
Outpatient		222 (70.7)
Reasons for Hospital Visit/Follow-up		
Delivery purposes		270 (86.0)
Other illnesses		44 (14.0)
Psychiatric Illnesses		
No psychiatric illness		298 (95.0)
Major depressive disorder		2 (0.6)
Anxiety disorder		8 (2.5)
More than one psychiatric disorder		6 (1.9)
Medical Conditions		
No medical condition		250 (79.6)
Metabolic-related		27 (8.6)
Others		37 (11.8)

**Table 2** presents both the binary and frequency distribution of adverse childhood experiences (ACEs) among the pregnant patients in the study. The binary component of the data reflects whether each ACE was experienced at least once during childhood, while the frequency component indicates how often these experiences occurred.

**Table 2:** Prevalence of adverse childhood experiences (ACEs) among participants at HCTM by ACE domains (binary vs. frequency scoring).

ACE Domain	Binary (%)	Frequency (%)
Physical abuse	Yes (11.5)	1.3
Emotional abuse	Yes (17.2)	1.9
Sexual abuse	Yes (4.8)	4.1
Substance use	Yes (1.1)	1.1
Incarceration	Yes (0.3)	0.3
Parental psychopathology	Yes (1.9)	1.6
Exposure to violence	Yes (26.1)	9.6
Parental divorce	Yes (29.3)	27.4
Emotional neglect	Yes (15.6)	15.6
Physical neglect	Yes (23.2)	5.1
Bullying	Yes (10.2)	0.3
Community violence	Yes (27.4)	0.6
Collective violence	Yes (2.2)	2.2

The highest prevalence was observed for parental separation or divorce, with 29.3% of participants reporting this experience, of which 27.4% reported it with higher frequency having undergone this event on more than one occasion. Any household member treated violently was reported by 26.1% of participants, with 9.6% reporting frequent occurrences. Community violence followed closely, with 27.4% reporting exposure, but only 0.6% experiencing it frequently. Emotional neglect had a prevalence of 15.6% in both binary and frequency scoring, indicating that this form of neglect was equally prevalent and recurrent among participants. Physical neglect was reported by 23.3% of participants, but only 5.1% experienced it with higher frequency. Physical abuse was reported by 11.5% of participants, with 1.3% reporting frequent occurrences. Emotional abuse affected 17.2% of participants, with 1.9% reporting it with higher frequency. Bullying was reported by 10.2% of participants, but only 0.3% reported it occurring frequently. Contact sexual abuse was reported by 4.8%, with 4.1% experiencing it frequently. Lower prevalence was found for other adversities: someone chronically depressed, mentally ill, institutionalized, or suicidal was reported by 1.9% and 1.6%, while alcohol and or drug abuse in the household was reported by 1% for both binary and frequency scores. Incarcerated household members were reported by 3%, with 0.3% reporting this experience with higher frequency. Contact sexual abuse was reported by 4.8% of participants, with 4.1% experiencing it frequently. Other adversities such as household mental illness, substance abuse, incarceration, and collective violence were reported at lower rates, ranging from 1% to 3%, with frequent occurrences being notably rare. Collective violence typically refers to experiences of violence that occur in the context of group conflict or widespread societal violence including wars, terrorism, political or ethnic conflicts, genocide, repression, disappearances, torture and organized violent crime such as banditry and gang warfare. This could include witnessing or experiencing violence from organized groups, militias, or during periods of civil unrest.

**Figure 1:**
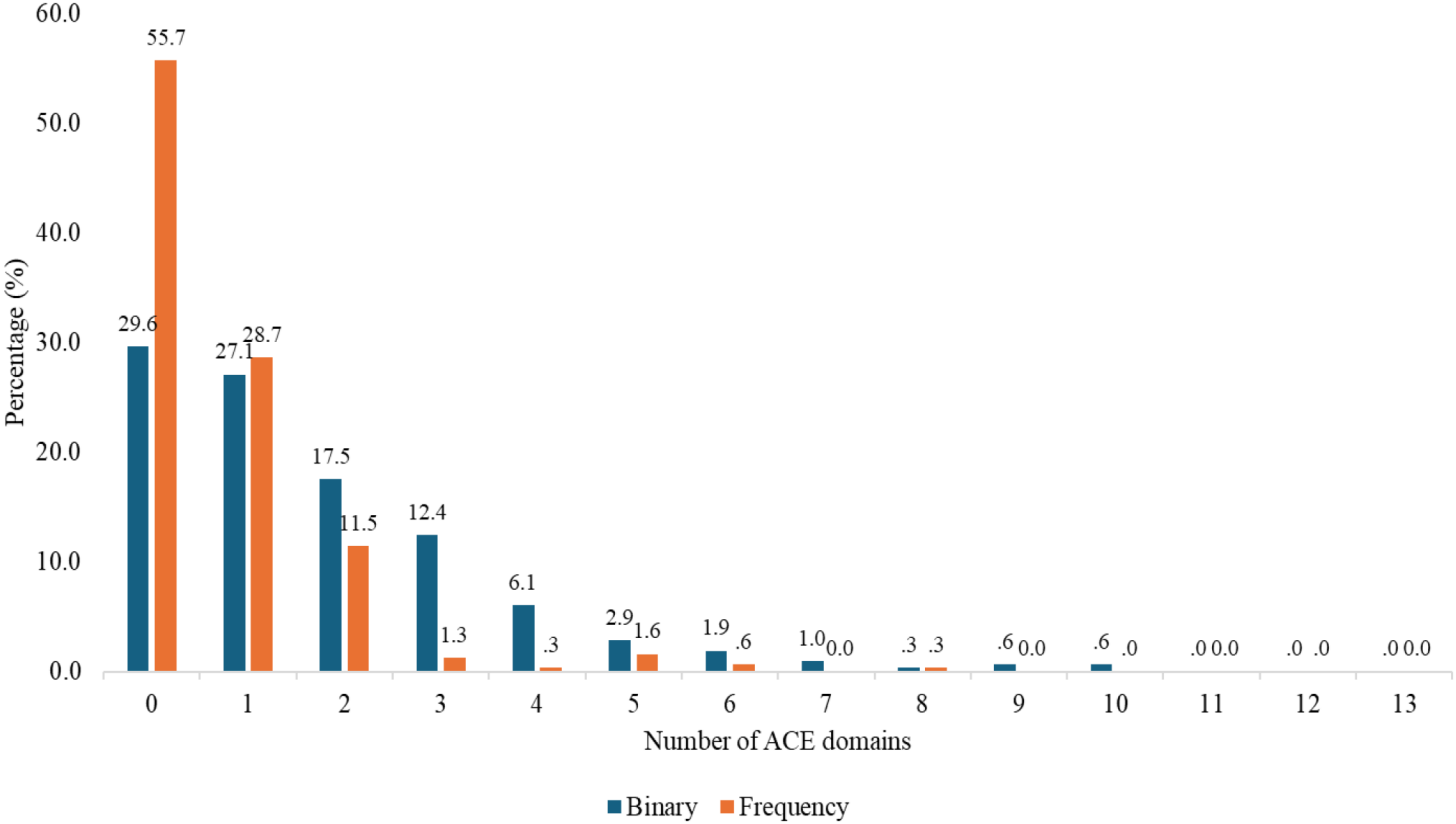
Distribution of participants by number of ACE domains experienced.

**Figure 1** presents the distribution of patients based on the number of ACE domains they reported experiencing. The distribution of participants according to the number of ACE domains experienced revealed notable patterns in both binary and frequency scores.

Approximately 29.6% of participants reported zero ACEs on the binary scale, indicating they had never experienced any adverse childhood experiences. Additionally, 55.7% of participants reported no frequent exposure to ACEs, meaning they either had never experienced ACEs or had experienced them infrequently. Notably, the 29.6% of participants who reported zero ACEs on the binary scale are included in the 55.7% reporting no frequent exposure, as individuals with no ACEs fall within this category. About 70% of the participants have ACEs. 27.1% reported having had just one ACE and 17.5% reported two, though the frequency of these experiences was lower, particularly for those with two ACEs (11.5%). Exposure to four or more ACE domains was less common, with only 13.4% of participants (combining those with four, five, six, and more ACEs) experiencing this level of adversity. The frequency of these higher-level exposures was also low, with fewer than 3% of participants reporting frequent exposure to four or more ACEs. These findings suggest that while a large proportion of participants experienced relatively limited ACE exposure (one to two domains), exposure to four or more ACEs was far less prevalent.

The overall prevalence of women at risk of depression in this study was found to be 13.7%, with a 95% confidence interval ranging from 9.9% to 17.5%.

**Table 3** presents the demographic, pregnancy, and clinical characteristics stratified by ACE score groupings (0, 1, 2, 3, ≥4) among the participants (n = 314). To identify variables significantly associated with the ACE score groupings, we assessed their p-values. Variables with p-values < 0.25 were shortlisted for inclusion in the multivariable analysis as potential confounders. The analysis of demographic, pregnancy, and clinical characteristics across ACE score groupings revealed no statistically significant differences for any of the examined variables. Specifically, age (p = 0.479), race (p = 0.609), occupation (p = 0.079), household income (p = 0.592), education level (p = 0.253), number of previous pregnancies (p = 0.325), planned pregnancy (p = 0.438), and trimester (p = 0.325) all had p-values greater than 0.05, indicating no significant variation across the ACE score categories.

**Table 3:** Demographic, pregnancy, and clinical characteristics stratified by ACE score groupings (n=314).

Characteristics	ACE score groupings					
	0	1	2		≥ 4	p-value
Age (n = 314)	31.7 (4.47)	31.6 (4.52)	32.5 (4.61)	30.7 (5.12)	31.5 (4.75)	0.479 ^b^
Race (n = 314)						0.609 ^a^
Malay	80 (30.9)	72 (27.8)	43 (16.6)	30 (11.6)	34 (13.1)	
Other races	13 (23.6)	13 (23.6)	12 (21.8)	9 (16.4)	8 (14.5)	
Occupation (n = 314)						0.079 ^a^
Employed	71 (31.4)	61 (27.0)	36 (15.9)	33 (14.6)	25 (11.1)	
Unemployed	22 (25.0)	24 (27.3)	19 (21.6)	6 (6.8)	17 (19.3)	
Household income (n = 314)						0.592 ^a^
< RM2500	17 (29.8)	15 (26.3)	10 (17.5)	5 (8.8)	10 (17.5)	
RM2501-RM4500	32 (29.4)	30 (27.5)	20 (18.3)	13 (11.9)	14 (12.8)	
RM4501-RM6500	22 (34.9)	20 (31.7)	10 (15.9)	6 (9.5)	5 (7.9)	
RM6501-RM8500	15 (34.1)	10 (22.7)	4 (9.1)	7 (15.9)	8 (18.2)	
> RM8500	7 (17.1)	10 (24.4)	11 (26.8)	8 (19.5)	5 (12.2)	
Education level (n = 314)						0.253 ^a^
≤ Secondary	18 (24.3)	18 (24.3)	18 (24.3)	12 (16.2)	8 (10.8)	
> Secondary	75 (31.3)	67 (27.9)	37 (15.4)	27 (11.3)	34 (14.2)	
Number of previous pregnancies (n = 314)						0.325 ^a^
0	21 (28.4)	21 (28.4)	10 (13.5)	8 (10.8)	14 (18.9)	
1	25 (34.7)	24 (33.3)	10 (13.9)	7 (9.7)	6 (8.3)	
2	22 (33.3)	17 (25.8)	12 (18.2)	9 (13.6)	6 (9.1)	
3	12 (22.2)	14 (25.9)	15 (27.8)	8 (14.8)	5 (9.3)	
≥ 4	13 (27.1)	9 (18.8)	8 (16.7)	7 (14.6)	11 (22.9)	
Planned pregnancy (n = 314)						0.438 ^a^
No	36 (25.5)	38 (27.0)	24 (17.0)	20 (14.2)	23 (16.3)	
Yes	57 (32.9)	47 (27.2)	31 (17.9)	19 (11.0)	19 (11.0)	
Trimester (n = 314)						0.325 ^a^
1	3 (23.1)	38 (27.0)	24 (17.0)	20 (14.2)	23 (16.3)	
2	18 (33.3)	47 (27.2)	31 (17.9)	19 (11.0)	19 (11.0)	
3	72 (29.1)	85 (27.1)	55 (17.5)	39 (12.4)	42 (13.4)	

^a^Pearson Chi-square test, ^b^One-way ANOVA.

**Table 4** illustrates the factors associated with depression risk among participants at HCTM, demonstrating a significant association between ACE score groupings and depression risk. The p-value for ACE score groupings was less than 0.001 (p < 0.01), indicating a strong association, with a higher percentage of women in the higher ACE score categories (3 and ≥ 4) reporting depression. In contrast, other factors did not show statistically significant associations with depression risk.

**Table 4:** Factors associated with depression risk among participants at HCTM (n=314).

Variables	Risk of depression		Test-statistic	p-value
	No	Yes		
ACE score groupings (n=314)			31.977 ^a^	< 0.001**
0	85 (91.4)	8 (8.6)		
1	80 (94.1)	5 (5.9)		
2	47 (85.5)	8 (14.5)		
3	34 (87.2)	5 (12.8)		
≥ 4	25 (59.5)	17 (40.5)		
Age (n=314)	31.7 (4.65)	31.5 (4.53)	0.271 ^b^	0.786
Race (n=314)			2.326 ^a^	0.127
Malay	220 (84.9)	39 (15.1)		
Other races	51 (92.7)	4 (7.3)		
Occupation (n=314)			2.083 ^a^	0.149
Employed	199 (88.1)	27 (11.9)		
Unemployed	72 (81.8)	16 (18.2)		
Household income (n=314)			2.370 ^a^	0.668
< RM2500	49 (86.0)	8 (14.0)		
RM2501-RM4500	91 (83.5)	18 (16.5)		
RM4501-RM6500	54 (85.7)	9 (14.3)		
RM6501-RM8500	39 (88.6)	5 (11.4)		
> RM8500	38 (92.7)	3 (7.3)		
Education level (n=314)			0.112 ^a^	0.738
≤ Secondary	63 (85.1)	11 (14.9)		
> Secondary	208 (86.7)	32 (13.3)		
Number of previous pregnancies (n=314)			5.007 ^a^	0.287
0	64 (86.5)	10 (13.5)		
1	65 (90.3)	7 (9.7)		
2	59 (89.4)	7 (10.6)		
3	46 (85.2)	8 (14.8)		
≥ 4	37 (77.1)	11 (22.9)		
Planned pregnancy (n=314)			3.528 ^a^	0.060
No	116 (82.3)	25 (17.7)		
Yes	155 (89.6)	18 (10.4)		
Trimester (n=314)			2.774 ^c^	0.248
1	11 (84.6)	2 (15.4)		
2	43 (79.6)	11 (20.4)		
3	217 (87.9)	30 (12.1)		

^a^Pearson Chi-square, ^b^Independent t-test, ^c^Fischer’s Exact Test, **p < 0.01.

**Table 5** presents the association of ACE score groupings with depression risk among pregnant women (n=314) across three models. Model 1 shows the unadjusted odds ratios for the relationship between ACE score groupings and depression risk. In this model, pregnant women with higher ACE scores (1, 2, 3, and ≥ 4) exhibited varying odds of depression compared to those with an ACE score of 0 (reference group), with the odds of depression increasing as ACE score groupings increased. Specifically, the unadjusted odds ratio for women with ≥ 4 ACE domains was 7.23 (95% CI = 2.79, 18.71, p < 0.001), indicating a significantly higher odds of depression. Model 2 adjusts for the total ACE score (0–13), treated as a continuous variable and the results remained consistent, with the odds ratio for those with ≥ 4 ACEs being 4.96 (95% CI = 1.47, 17.02, p = 0.011), suggesting that even after adjusting for the frequency of ACEs, the association with depression risk remained significant. Model 3 further adjusted for potential confounders whereby variables with p-values < 0.25 were shortlisted for inclusion in the multivariable analysis, including race (Malay vs. other races), occupation (employed vs. unemployed), pregnancy planning (planned vs. unplanned), and trimester (1 versus 2 versus 3). In this final model, the adjusted odds ratio for women with ≥ 4 ACEs was 4.33 (95% CI = 1.23, 15.22, p = 0.022), confirming a significant association between higher ACE scores and increased depression risk. This model showed a slight improvement in Nagelkerke’s R square (0.188), indicating a modest increase in explanatory power after adjusting for confounders. Overall, despite adjusting for potential confounders, the ACE score groupings remained significantly associated with depression risk, with pregnant women in the highest ACE group (≥ 4) having over four times the odds of experiencing depression compared to those with no ACEs.

**Table 5:** Association between ACE score groupings and depression risk among participants at HCTM (n=314).

Models	ACE score groupings	Adjusted Odds ratio (95% CI)	p-value	Nagelkerke’s R square
1 (n=314)	0 (ref)			0.145
	1	0.664 (0.209, 2.115)	0.488	
	2	1.809 (0.637, 5.131)	0.265	
	3	1.562 (0.477, 5.116)	0.461	
	≥ 4	7.225 (2.791, 18.706)	<0.001**	
2 (n=314)	0 (ref)			0.150
	1 2	0.621 (0.193, 1.995) 1.606 (0.549, 4.700)	0.424 0.387	
	3	1.308 (0.377, 4.542)	0.672	
	≥ 4	4.960 (1.446, 17.019)	0.011*	
3 (n=314)	0 (ref)			0.188
	1	0.595 (0.183, 1.937)	0.388	
	2	1.597 (0.537, 4.750)	0.400	
	3	1.300 (0.363, 4.653)	0.687	
	≥ 4	4.326 (1.230, 15.217)	0.022*	

CI, Confidence interval; ref, Reference group; *p < 0.05; **p < 0.01.

1 = Unadjusted.

2 = Adjusted for ACE score (Frequency).

3 = Adjusted for race (Malay vs other races), Occupation (Employed vs unemployed), Planned pregnancy (No vs Yes), and trimester (First vs second vs third).

**Table 6** presents the association between individual ACE domains and depression risk among pregnant women, with the odds ratios adjusted for the same confounders as in **Table 5**, except for the ACE frequency score. Instead, each ACE domain was adjusted for its corresponding frequency score. The analysis reveals that five out of the 13 ACE domains were significantly associated with an increased risk of depression. Specifically, physical abuse (aOR = 5.08, 95% CI = 2.20, 11.73, p < 0.001), emotional abuse (aOR = 3.54, 95% CI = 1.66, 7.58, p = 0.001), household member treated violently (aOR = 4.40, 95% CI = 1.94, 9.96, p < 0.001), physical neglect (aOR = 2.61, 95% CI = 1.26, 5.60, p = 0.010), and community violence (aOR = 2.16, 95% CI = 1.08, 4.33, p = 0.03) were all significantly related to depression risk. Several ACE domains could not be computed due to low numbers of “Yes” responses, including alcohol and/or drug abuser in the household, incarcerated household member, someone chronically depressed or suicidal and collective violence.

**Table 6:** Association of ACE domains with depression risk among participants at HCTM (n=314).

ACE Domain	Adjusted Odds Ratio (95% CI)	p-value
Physical abuse	5.08 (2.20, 11.73)	< 0.001**
Emotional abuse	3.54 (1.66, 7.58)	0.001**
Contact sexual abuse	3.82 (0.23, 64.54)	0.35
Household member with alcohol and/or drug abuse	—	†
Incarcerated household member	—	†
Household member mentally ill/institutionalized/suicidal	—	†
Household member treated violently	4.40 (1.94, 9.96)	< 0.001**
One or no parents, parental separation, or divorce	1.11 (0.54, 2.26)	0.777
Emotional neglect	1.84 (0.82, 4.16)	0.140
Physical neglect	2.65 (1.26, 5.59)	0.010*
Bullying	2.35 (0.95, 5.80)	0.064
Community violence	2.16 (1.08, 4.33)	0.030*
Collective violence	—	†

† Unable to compute due to low number of “Yes” responses.

*Significant at p < 0.05, **Significant at p < 0.01.

All models adjusted for race (Malay vs other races), Occupation (Employed vs unemployed), Planned pregnancy (No vs Yes), trimester (First vs second vs third), and corresponding ACE domains (Frequency) (e.g., physical abuse [frequency]).

## 4 Discussion

This study aimed to investigate the relationship between ACEs and the risk of depression among pregnant women. We hypothesized that women with a history of ACEs would have a higher likelihood of developing antenatal depression. Our findings confirm this hypothesis, revealing significant associations between various types of ACEs and an increased prevalence of antenatal depression. These results offer valuable insights into the potential long-term effects of childhood adversities on maternal mental health, emphasizing the important connection between ACEs and the risk of depression during pregnancy. In addition to ACEs, we also examined other sociodemographic factors that may contribute to antenatal depression. The discussion that follows synthesizes these findings, reflecting on the overall frequency of antenatal depression and ACEs and the observed associations. The overall frequency of women at risk of depression in our study was found to be 13.7%, which closely aligns with another study conducted among women in Sabah, reporting a prevalence rate of 13.8% (28). Additionally, a separate study focusing on the east and west coasts of Malaysia identified a prevalence rate of 12.2% (3). On a global scale, the findings are also comparable, as the pooled prevalence of major antenatal depression across 72 studies with 79 reports was determined to be 15.0% (6).

In our study, approximately 70.4% of participants reported experiencing at least one form of ACE, a finding consistent with existing literature. For instance, a local study reported a prevalence of 63.8% for ACEs (22), while in another study done in Singapore, it was reported that the lifetime prevalence of ACEs in their sample was 63.9% (14). Similarly, in a large-scale US-based study involving 248,934 participants found that 61.55% had encountered at least one ACE (29).

These findings underscore the widespread nature of ACEs, suggesting that a significant proportion of pregnant women may have a history of childhood adversities, thereby highlighting the growing body of evidence regarding the prevalence of ACEs and their potential implications for maternal health.

Parental separation or divorce was notably prevalent, affecting nearly one-third of participants. This was followed by exposure to community violence (27.4%), witnessing household members being treated violently (26.1%), and physical neglect (23.2%). These findings are consistent with those of a local study by Asyraf et al. (2021), which identified growing up with one or no parent as the most common ACE (prevalence 24.4%), followed by physical neglect (24%) and exposure to community violence (20.4%) (22). Similarly, our results align with a study conducted in Norway, which surveyed 8,199 participants. In this study, parental divorce emerged as the most common ACE (29.5%), followed by witnessing violence (22.1%) and experiencing physical abuse (14.7%) (30). These comparisons highlight the consistency of ACE prevalence across different populations, suggesting that certain forms of childhood adversity, such as parental separation and exposure to violence, are widespread issues that transcend geographical and cultural contexts. The high prevalence of ACEs related to parental separation and single-parent households in our study may be attributed to the rising divorce rate in Malaysia. As the divorce rate increases, a greater number of children are raised in single-parent families, which can negatively impact their childhood experiences. Parenting stress, which is commonly faced by single parents, can contribute to adverse outcomes for children, including an elevated risk of maltreatment. Furthermore, children in single-parent families, particularly those who experience significant stress from the primary caregiver, are at a higher risk of developing psychological issues such as depression and behavioral problems (31, 32). The growing number of single-parent households emphasizes the importance of understanding the psychological consequences for children and highlights the need for effective interventions to support children in these family structures.

The frequency of ACEs was also notable, with certain adversities, such as household violence and community violence, reported frequently by a smaller but significant portion of the sample. This indicates that not only is childhood adversity widespread, but the recurrence of certain experiences (e.g., household violence) can exacerbate their potential long-term psychological effects.

Our analysis found a significant association between ACE score groupings and depression risk among pregnant women. In the unadjusted model (**Table 5**, Model 1), we observed that higher ACE scores were linked to a greater likelihood of depression. Participants with one to three ACEs did not show a significant increase in depression risk compared to those with no ACEs. This may reflect a threshold effect, where mental health risks become more pronounced only at higher levels of adversity (≥4 ACEs). Additionally, protective factors such as resilience or social support may buffer the effects of lower ACE exposure. These findings align with existing literature supporting the ≥4 ACE cut-off as clinically meaningful. Notably, women with four or more ACE domains exhibited a more than sevenfold increase in the odds of depression (OR = 7.23, 95% CI = 2.79, 18.71, p < 0.001). This finding remained robust in both the adjusted models, with the association persisting even after controlling for potential confounders in Model 3 (aOR = 4.60, 95% CI = 1.32, 16.02, p = 0.017). These findings are consistent with several studies which demonstrated a strong, dose – response relationship between the ACE score and depressive disorders (7, 33, 34).

In addition to examining overall ACE scores, we explored the relationship between individual ACE domains and depression risk. Specifically, five out of the 13 ACE domains—physical abuse, emotional abuse, household member treated violently, physical neglect, and community violence—were found to be significantly associated with an increased risk of depression. The strongest association was observed for physical abuse, with an adjusted odds ratio of 5.08, indicating that pregnant women who experienced physical abuse during childhood had more than five times the odds of experiencing depression compared to those who did not experience such abuse. Similarly, emotional abuse and household member treated violently also demonstrated significant associations with depression, with aORs of 3.54 and 4.40, respectively. These findings align with previous research showing that childhood maltreatment, particularly abuse and exposure to violence, significantly increases the risk of mental health issues later in life, including depression (35). The psychological and emotional consequences of such experiences, such as feelings of powerlessness, low self-worth, and chronic stress, may contribute to the heightened vulnerability to depression in adulthood, particularly during the sensitive period of pregnancy.

Physical neglect was also found to be significantly associated with depression risk (aOR = 2.65), though the strength of the association was lower compared to the abuse-related domains. This shows that neglect, while potentially less overt than abuse, may still have profound long-term effects on mental health, potentially due to its impact on attachment, emotional regulation, and overall psychosocial development (36).

Community violence, though less frequently studied in the context of ACEs, was also significantly linked to depression risk (aOR = 2.16), supporting the idea that exposure to violence in the broader environment can contribute to long-term mental health challenges. The high rate of community violence observed in the study population may be attributed to a combination of structural and social challenges present within the Malaysian setting. Community violence—which encompasses incidents such as assaults, physical altercations, gang activity, robberies, and localized criminal behavior—is often more prevalent in economically disadvantaged or densely populated urban areas. In such environments, factors like income inequality, unemployment, limited educational opportunities, and inadequate access to mental health services may heighten vulnerability to violence. These underlying social conditions increase the likelihood of individuals encountering or witnessing violence in their immediate surroundings, particularly within marginalized or underserved communities.

Interestingly, while 10.2% of participants reported experiencing bullying, only 0.3% indicated that it occurred frequently. This discrepancy raises important considerations regarding the severity and impact of bullying. It could suggest that although bullying is relatively common, the intensity or frequency of these experiences may not be as high for most individuals. Alternatively, it may indicate that participants perceive bullying as less frequent but still significant, or they may have been reluctant to label their experiences as ‘frequent’ despite its occurrence. Further research could help clarify the nature of these experiences and their lasting effects on mental health, especially considering that even infrequent bullying can have substantial emotional and psychological consequences.

While parental divorce was not significantly associated with antenatal depression in this sample, several factors may help explain this finding. Antenatal depression is a multifactorial condition, influenced by biological, psychological, and social determinants, and it may not be solely explained by childhood experiences such as parental divorce. It is possible that other adverse childhood experiences (ACEs) in the sample had a more significant impact on antenatal depression, potentially overshadowing the effects of parental divorce.

Additionally, the timing, nature, and context of parental divorce could be important factors. For instance, the age at which the divorce occurred, the perceived severity of the event, or whether the divorce was recent, could influence its emotional impact. It is also conceivable that some participants may have experienced parental divorce at a later developmental stage, with less emotional impact compared to those who experienced it at a younger age.

Furthermore, protective factors such as strong social support or resilience could have mitigated the negative effects of parental divorce, reducing its potential impact on antenatal depression.

Other ACE domains, such as contact sexual abuse and emotional neglect were not found to have a significant association with depression risk in this study. This may be due to various factors, including differences in how these experiences are perceived or remembered by individuals, or the complex nature of their psychological effects. It could also reflect the limitations of this study’s methodology. For instance, the low number of responses for some domains, such as alcohol or drug abuse in the household, incarcerated household members, and chronic depression or suicidal behaviors, may have limited the power to detect significant associations. The inability to compute these domains further emphasizes the importance of considering the completeness of ACE data in research and the challenges associated with capturing all forms of adversity that could contribute to mental health outcomes. However, it is important to note that some of these ACE domains—such as contact sexual abuse and parental separation or divorce—have been linked to depression in other studies (37, 38), suggesting that these associations may vary depending on the population studied, the methods of assessment, or the specific context of the study.

Overall, these findings suggest that specific ACE domains, particularly those involving abuse, neglect, and violence, have a significant and lasting impact on depression risk among pregnant women. This highlights the need for targeted early diagnosis, support, and therapeutic interventions to improve both maternal and fetal well-being.

Efforts to prevent ACEs must take a comprehensive, multi-level approach that tackles contributing factors at the personal, familial, community, and institutional levels. In Malaysia, this involves reinforcing existing child protection systems, enhancing the early detection of children at risk, and improving access to mental health services, parenting support, and safe learning environments. Raising public awareness through education campaigns and reducing barriers to seeking help are also crucial steps. Community-focused strategies, including youth empowerment activities and neighborhood crime prevention programs, can help lower the incidence of community and collective violence. On a broader scale, effective prevention relies on coordinated efforts among healthcare, education, welfare, and law enforcement agencies to build a trauma-informed support network. Strengthening these preventive measures and protective resources has the potential to greatly improve long-term outcomes for children facing adversity in the Malaysian context.

Following ACEs screening, appropriate care should be provided, including psychosocial support such as counseling and trauma-focused therapies like Cognitive Behavioral Therapy (CBT), which can help address emotional challenges. Psychiatric care may be necessary for those exhibiting symptoms of anxiety, depression, or PTSD, with selective serotonin reuptake inhibitors (SSRIs) being a potential treatment option, though with careful consideration during pregnancy. Referral to social services is crucial for addressing financial, housing, or childcare needs, while mindfulness- based stress reduction programs can assist in managing stress and improving emotional regulation. A collaborative care approach, involving obstetricians, mental health professionals, and social workers, is essential for holistic care and regular follow-up. Additionally, providing preventative education on the effects of ACEs, self-care, and the importance of seeking help can empower women to take proactive steps in managing their health and mitigating the impact of ACEs on their pregnancy and overall well-being.

The high response rate of 96.9% is a key strength of our study, reflecting strong engagement and providing a solid foundation for our findings. In addition to identifying prevalent risk factors associated with antenatal depression, our investigation delves into the impact of a history of adverse childhood experiences (ACEs) on maternal mental health. We utilized the globally recognized Adverse Childhood Experiences International Questionnaire (ACE-IQ),

which has been translated into Malay and validated for use in our population. This study is among the first to explore the relationship between ACEs and antenatal depression within this population, offering valuable insights into local antenatal mental health trends.

However, despite these strengths, the study has several limitations. The cross-sectional design of the study restricts our ability to establish causal relationships, while the reliance on self- reported data introduces the potential for recall bias, particularly for participants with higher ACE scores. Additionally, the study’s sample size may have limited statistical power, potentially preventing the detection of significant associations in some ACE domains. The lack of randomization in our sampling also introduces the possibility of selection bias, as participants may not fully represent the broader population of pregnant women. Furthermore, the use of convenience sampling from antenatal clinics and wards may contribute to sampling bias, limiting the generalizability of our findings.

The low Nagelkerke’s R² value of 0.184 suggests that the model accounts for only a modest portion of the variance in antenatal depression. This could be due to unmeasured factors, such as intimate partner violence (IPV), social support, or substance use, which were not included in the model. Additionally, while maternal age and education level—known predictors of perinatal mental health were collected, they were not included in the final model. This may have limited our ability to fully adjust for potential confounders. The study also lacks verification of certain clinical data, such as obstetric complications, which could have influenced depression risk. To address these limitations, future research should incorporate more comprehensive data and employ longitudinal designs to explore the long-term effects of ACEs on maternal mental health.

In summary, while this study provides important insights into the relationship between ACEs and antenatal depression, it also highlights the need for further research to explore these issues in greater depth. Further research is needed to explore the mechanisms underlying the associations between adverse childhood experiences (ACEs) and maternal mental health, as well as to investigate other potential ACE domains that may affect maternal well-being. Additionally, including a more diverse study population could provide a broader understanding of how ACEs affect depression risk across different demographic and cultural contexts, enhancing the generalizability of the results. It is also crucial to recognize that the timing of these ACEs in relation to brain development can influence the extent and nature of the mental health outcomes. Further research should focus on understanding how ACEs affect the brain at various developmental stages—whether during early childhood, adolescence, or even later in life—since the brain undergoes significant changes and maturation at each stage. This deeper understanding could help develop targeted interventions that address the specific needs of individuals at different stages of brain development, ultimately improving mental health outcomes for those affected by ACEs.

## 5 Conclusion

The prevalence of women at risk for depression at HCTM, as measured by the EPDS, is 13.7%. This study highlights the lasting impact of ACEs on maternal mental health, with physical abuse, emotional abuse, household violence, physical neglect and community violence showing strong associations with depression risk. Notably, a dose-response relationship was observed, with depression risk increasing as the number of ACEs rose, particularly for women reporting four or more ACEs, who faced more than a fourfold increase in depression risk. This study reinforces the importance of integrating depression screening into antenatal services, particularly in those with a history of childhood adversity.

## Data Availability

The raw data supporting the conclusions of this article will be made available by the authors, without undue reservation.
